# Comprehensive annotation of microRNA expression profiles

**DOI:** 10.1186/1471-2156-14-120

**Published:** 2013-12-20

**Authors:** Bo Sun, Fei Yang, Fei-Hu Hu, Ning-Ping Huang, Zhong-Dang Xiao

**Affiliations:** 1 State Key Laboratory of Bioelectronics, School of Biological Science and Medical Engineering, Southeast University, Nanjing 210096, P. R. China

**Keywords:** MicroRNA, Microarray, Deep sequencing, Comprehensive annotation

## Abstract

**Background:**

MicroRNAs (miRNAs) regulate many biological processes by post-translational gene silencing. Analysis of miRNA expression profiles is a reliable method for investigating particular biological processes due to the stability of miRNA and the development of advanced sequencing methods. However, this approach is limited by the broad specificity of miRNAs, which may target several mRNAs.

**Result:**

In this study, we developed a method for comprehensive annotation of miRNA array or deep sequencing data for investigation of cellular biological effects. Using this method, the specific pathways and biological processes involved in Alzheimer’s disease were predicted with high correlation in four independent samples. Furthermore, this method was validated for evaluation of cadmium telluride (CdTe) nanomaterial cytotoxicity. As a result, apoptosis pathways were selected as the top pathways associated with CdTe nanoparticle exposure, which is consistent with previous studies.

**Conclusions:**

Our findings contribute to the validation of miRNA microarray or deep sequencing results for early diagnosis of disease and evaluation of the biological safety of new materials and drugs.

## Background

MicroRNAs (miRNAs) are short ribonucleic acid (RNA) molecules with an average length of 22 nucleotides (nt), which exhibit higher stability than messenger RNAs (mRNAs) [1,2]. They are post-transcriptional regulators that bind to complementary sequences on target mRNA transcripts, usually resulting in translational repression or target degradation and gene silencing [3-5]. MiRNAs regulate numerous biological processes, including cell viability, proliferation, development and differentiation [1,2]. Similar to mRNA microarray techniques, methods for studying miRNA expression profiles have been developed including deep sequencing techniques [6,7]. In addition to the evaluation of the stability of miRNA during sample processing, the assessment of differential miRNA expression profiles has been identified as a reliable method for the investigation of mRNAs, proteins and mechanistic pathways involved in particular biological processes, such as differentiation, carcinogenesis and cytotoxicity. Indeed, methods have been developed to validate miRNA microarray or deep sequencing data for biological research purposes, such as the prediction of miRNA function and activity, interaction of miRNA and mRNAs and the investigation of miRNA regulatory influences on sub-pathways [8-10].

**Figure 1:**
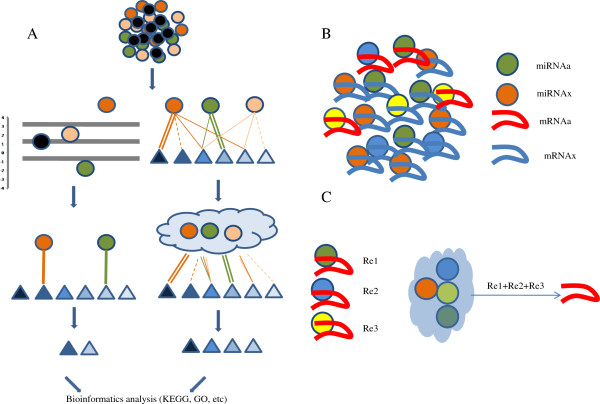
**The global repression effect of a miRNA expression profile on a specific miRNA.** Generally, for miRNA microarray or deep sequencing data analysis, miRNAs (circle) with significantly changed expression profiles are selected through fold-change analysis. The target mRNAs (triangle) of the selected miRNA are predicted by miRNA target prediction tools such as miRanda **(A)**. Theoretically, a mRNA may be targeted by several miRNAs with different context scores, which are regarded as the repression ratio score of a miRNA for its target **(B)**. Thus, the total repression ratio score of the total miRNA expression profile for a specific mRNA can be calculated based on the combined repression effects of the miRNAs on their target mRNA (**C**, see formula (1)).

Generally, miRNAs shown to be expressed at significantly different levels by miRNAs array/sequencing are selected out through fold-change analysis. The target genes of selected miRNA are predicted by tools such as PicTar, myMIR, TargetScan and miRanda [11-15]. Subsequently, these predicted genes are enriched in KEGG pathway or Gene Ontology (GO) analyses [16-18]. The KEGG pathway database records networks of molecular interactions in cells and the GO analysis provides the ontology of defined terms that represent gene product properties. Three domains are covered by GO: biological processes, molecular functions and sets of molecular events with a defined beginning and end (Figure 1A).

However, miRNAs exhibit broad specificity and may target several mRNAs in a given cell. The methods for miRNA target prediction had been intensively studied [19-22] and the tools available generate several scores for different potential targets of a particular miRNA. This reflects the complex regulatory networks of miRNAs and mRNAs. Thus, the repression of a particular mRNA by a given set of miRNAs is mediated by the combined effects of each individual miRNA on this common target mRNA [6]. Due to this combinatorial repression effect, the abundance of each miRNA will also contribute to the repression capacity of a given set of miRNAs (Figure 1A).

Based on this study, a mathematical model has been proposed that can comprehensively predict genes that are effectively regulated by a given miRNA. This is achieved by integrating the effect of each miRNA on their target mRNA based on the combined repressive effects of the relevant miRNAs. This model can be used to elucidate the combined effects of a miRNA profile.

## Methods

### Data source

MiRNA microarray data from four Alzheimer’s disease subjects were obtained from the NCBI Gene Expression Omnibus (GEO), as reported previously [23]. In this study, postmortem human brain samples were obtained and RNA was extracted from parietal lobes of postmortem brains of Alzheimer’s disease patients and controls. The mRNA array measurements were performed at the UCLA microarray core using Affymetrix HG-U133 Plus 2.0 arrays. MicroRNAs were assayed by LC Sciences using a custom -Paraflo array containing probes for 470 miRNAs from Sanger miRBase and 419 miRNAs predicted by miRNAMap.

MiRNA deep sequencing data were obtained in a previous study that evaluated CdTe nanoparticle cytotoxicity [6]. In this study, NIH/3 T3 cells were exposed to cadmium telluride quantum dots (CdTe QD) to extract the small RNAs. After exposure to nanomaterials for 24 h, the cells were harvested with the trypsin to extract the miRNAs. Then, the small RNAs in a sample were converted into a double-stranded cDNA library. The results of SOLiD sequencing were in the form of nucleotide sequences and their coverage. The registered miRNAs were screened out by comparing them in GenBank (http://www.ncbi.nlm.gov/genbank/) and miRbase (http://www.mirbase.org/).

## Methods

### A mathematical model to evaluate the comprehensive repression rate of specific mRNAs using total miRNA expression profiles

The repression (*Re*) of mRNA is directly proportional to the inhibitory effect (*IE*) of miRNA. For instance, for a specific mRNA, mRNAα, regulated by specific miRNAs, the formula is as follows:

(1) RemRNAα∝∑i=anIEi−α

Where *IE*_*i-α*_indicate the inhibitory effect of a specific miRNA*i* (*i = a, b, c,……n*) to mRNA*α*.

For a particular miRNA, miRNA*a*, *IE*_*a*_ is directly proportional to the distribution of miRNA*a* (*DU*_*a*_) on its target mRNA*α* (represented as *DU*_*a-α*_ in the following formula) and the repression score *(RS*) of miRNA*a* for its target mRNA*α*, which can be obtained from miRanda or other tools:

(2) IEa−α∝DUa−α×RSa−α

*DU*_*a-α*_ is directly proportional to the abundance of miRNA*a* (*AU*_*a*_) in the given miRNA array or sequencing database and the ratio of miRNA*a* combined with mRNA*α* (*CO*_*a,α*_):

(3) DUa−α∝AUa×COa−α

A single miRNA*a* may target several mRNAs. By induction of *Pa* − *α* as the proportion of miRNA*a* combined with mRNA_*α*_, *COa* − *α* can be calculated as:

(4) $$\text{CO}a-\alpha =\frac{Pa-\alpha }{\sum_{i=\alpha }^{n}Pa-i}$$

Scoring in prediction tools is designed to reveal the ability of miRNA to bind complementary regions of mRNAs [24] and previous work has shown that contexts of 7-nt or 8-nt matches appear sufficient for miRNA-like regulation [25,26]. Studies have concluded that additional recognition features, such as pairing with the remainder of the miRNA, accessible mRNA structures and protein-binding sites are usually dispensable or occur so frequently that they impart little overall specificity [27]. Hence,

(5) Pa−α∝RSa−α

In formula (3), AU_*a*_ indicates the abundance of miRNAa in the given miRNA array or sequencing database. We designated *C*_*a*_ as the counts of miRNA*a* in the sequencing data (or the signal value in microarray data), and *TC*_*miRNAs*_ as the total counts (or the signal value for microarray analysis) of miRNA provided by the sequencing data. Thus, AU_*a*_ can be calculated as:

(6) $$\text{AU}a=\frac{\text{Ca}}{\text{TCmiRNAS}}$$

By combining formulae (3), (5) and (6), *DU*_*miRNAa*_ can be calculated as:

(7) $$\text{DU}a-\alpha \;\propto \;\left(\frac{\text{Ca}}{\text{TCmiRNAs}}\times \frac{\text{RS}a-\alpha }{\sum_{i=\alpha }^{n}\text{RS}a-i}\right)$$

Hence, according to formula (2), *IE*_*miRNAa*_ can be calculated as:

(8) $$\text{IE}a-\alpha \propto \frac{\text{Ca}\times {\left(\text{RS}a-\alpha \right)}^{2}}{\text{TCmiRNAs}\times \sum_{i=\alpha }^{n}\text{RS}a-i}$$

By combining formulae (8) and (1), and induction of *K* as a coefficient factor, the total regression of a given miRNA sequencing data to mRNA*α* can be calculated as:

(9) RemRNAα=K×∑i=anCa×RSi−α2TCmiRNAs×∑i=αnRSa−i

### Statistical analysis

*Z*-tests were performed to investigate differences in the repression of target mRNAs by miRNAs. *p0* was calculated using formula (10), which was used to investigate the null hypothesis.

(10) p0α=RemRNAαControl+RemRNAαTestTCmiRNAControl+TCmiRNATest

Here, *RemRNAα*_*Control*_ and *RemRNAα*_*Test*_ indicate the repression of mRNA*α* in the control and test groups, whereas *TCmiRNA*_*Control*_ and *TCmiRNA*_*Test*_ indicate the total counts of miRNA in the control and test groups.

Then, the *Z*-test was performed according to the following formula:

(11) Za=RemRNATest‒RemRNAαControlp0α1‒p0α1TCmiRNAControl+1TCmiRNATest

The null hypothesis was rejected at *Z*-values >2.58 or < −2.58, which indicated significant differences between the repression of mRNAs in the test and control groups.

## Results and discussion

### The global repression effect of a miRNA expression profile on a specific mRNA

It has been reported that an individual miRNA s may target different mRNAs. Indeed, context scores for ranking the predicted targets of each miRNA have been provided by previous studies [10,28,29]. In contrast, one mRNA may be targeted by several miRNAs with different context scores, which are represented by the repression ratio score of an individual miRNA for its target (Figure 1B) [30]. To validate miRNA microarray or deep sequencing results for the prediction of changes in proteins or mechanistic pathways, the total repression ratio score of the total miRNA expression profile for a specific mRNA was calculated based on the combined miRNA repression effects on their target mRNA (Figure 1C, see formula (1)).

The repression capacity of an individual miRNA on its target mRNA is affected by miRNA concentration and the repression score identified using programs such as PicTar. MiRNAs bind targets with different efficiencies and therefore, the distribution of an individual miRNA on the specific target should be considered. As scoring in prediction tools is designed to reveal the capacity of miRNA to bind complementary regions of mRNAs, the repression ability of a miRNA is directly proportional to the square of the repression score as shown in formula (9). Formula (9) shows that, for a given miRNA, the efficiency of binding to a given miRNA affects the repression capacity in an exponential manner. Based on the total repression effect of a given miRNA expression profile on a specific mRNA, the significantly regulated mRNA can be selected out by Z-tests for further analysis.

### Prediction of biological pathway and process regulation using miRNA expression profiles based on array data

In order to validate our method for identification of specific pathways or biological processes regulated by a given miRNA profile, the significantly regulated mRNA selected by our algorithm was enriched in KEGG pathways or GO terms.

Data from four Alzheimer’s disease subjects and one control subject were used in this study. In order to apply KEGG pathway or GO terms analyses for the precise and comprehensive elucidation of the effects of miRNAs on biological processes in Alzheimer’s disease, the repression effect of Alzheimer’s disease miRNA profiles on a specific mRNA were calculated according to the repression value obtained using miRNA target prediction tools and the interaction properties of miRNA and mRNA. Formula (9) gives a total repression score of a specific mRNA, which is directly proportional to the abundance of the related miRNA and the square of the repression value obtained using miRNA target prediction tools. For example, in sample S3, beta-catenin mRNA (ENTREZ_GENE_ID:1499; NM_001904) is regulated by several miRNAs with different repression scores given by miRanda, such as hsa-miR-139, hsa-miR-200a and hsa-miR-320. The total repression rate calculated using formula (9) was 10,296 in S3 compared with 5,096 in the control group. Based on the repression rate, a Z-test was performed for selection of mRNAs for further analysis.

**Figure 2:**
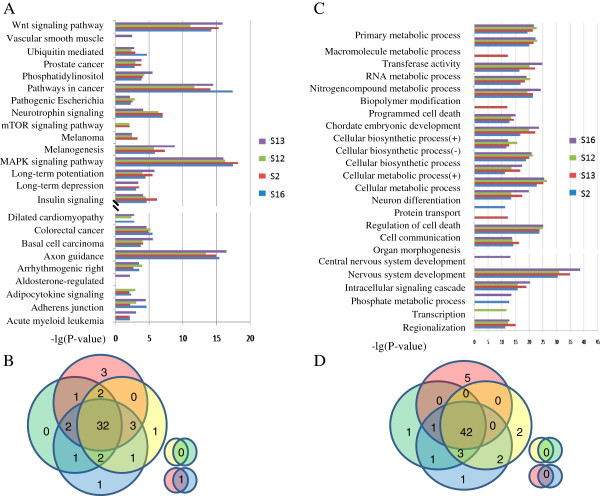
**KEGG pathways and GO terms significantly regulated by miRNAs in Alzheimer’s disease.** KEGG pathways significantly regulated by miRNAs are summarized in **(A)**. Most KEGG pathways were shared by all four Alzheimer’s disease samples (64%, 32/50) **(B)**. GO terms significantly regulated by miRNAs are summarized in **(C)**. Four Alzheimer’s disease samples shared most of these biological processes (72%, 42/58) **(D)**. (+) indicates the positive regulation while (−) indicates negative regulation.

**Figure 3:**
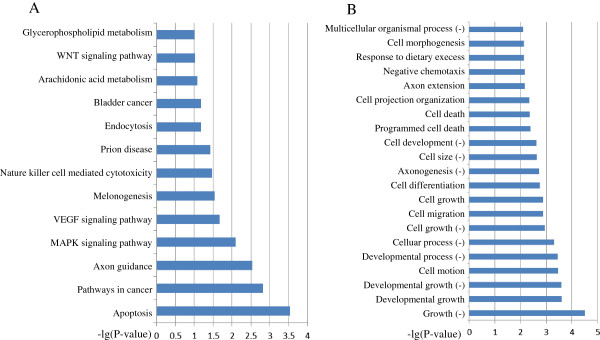
**KEGG pathways and GO terms significantly regulated by miRNAs after CdTe QD treatment.** SOLiD sequencing based miRNA expression profile data of the CdTe QD treated cells were applied to our algorithm. Selected mRNAs indicated to be under significant repression by miRNA profiling were enriched using KEGG pathways **(A)** and GO terms **(B)**. (+) indicates the positive regulation while (−) indicates negative regulation.

The selected mRNAs were enriched into KEGG by using the web-based GO analysis tool, DAVID and it was shown that the four Alzheimer’s disease samples shared most of the KEGG pathways that were found to be significantly altered under miRNA regulation (Figure 2A). Among these significantly regulated KEGG pathways, four pathways (WNT signaling pathway, MAPK signaling pathway, axon guidance and pathways involved in cancer) were highly regulated by miRNA in all of the four Alzheimer’s disease samples (Table 1). Other pathways, such as endocytosis, focal adhesion, neurotrophin signaling pathway and regulation of the actin cytoskeleton also showed a significant difference between the four Alzheimer’s disease samples and the control samples *(P <* 10^-5^). Studies have illustrated the close correlation of these pathways with Alzheimer’s disease [31-38]. Several other selected pathways *(P <* 0.01) are also shown in Figure 3. Most of these, such as gap junctions, have been reported in the studies on Alzheimer’s disease [39].

**Table 1:** The top 5 pathways that were most affected by the miRNAs in the four Alzheimer’s disease samples

**S2**		**S3**		**S12**		**S16**	
**KEGG pathways**	**-lg(P Value)**	**KEGG pathways**	**-lg(P Value)**	**KEGG pathways**	**-lg(P Value)**	**KEGG pathways**	**-lg(P Value)**
MAPK signaling	18.25	Axon guidance	16.54	MAPK signaling	16.29	Axon guidance	16.54
Wnt signaling	15.34	MAPK signaling	16.04	Axon guidance	13.41	MAPK signaling	16.04
Axon guidance	15.00	Wnt signaling	15.92	Cancer	11.70	Wnt signaling	15.92
Cancer	14.10	Cancer	14.55	Wnt signaling	11.15	Cancer	14.55
Endocytosis	8.85	Melano-genesis	8.82	Endocytosis	7.77	Melano-genesis	8.82

The GO provides the ontology of defined terms representing gene product properties. Among the three domains covered by the GO, biological processes, operations or sets of molecular events with either a defined beginning or end, can relevantly describe the functioning of integrated genes. The biological processes involved in Alzheimer’s disease were assessed based on the significantly regulated genes, using DAVID. Cellular processes were widely regulated in Alzheimer’s disease compared with the control group. Among these cellular processes, the nervous system development process was markedly altered in all four Alzheimer’s samples *(P <* 10^-25^). In this process, genes selected using our algorithm included presenilin (NM_000021), superoxide dismutase (NM_000384) and the oxytocin receptor (NM_000794), which have been reported to be involved in Alzheimer’s disease [40-42]. Other processes implicated in Alzheimer’s disease are also presented in Figure 2A. Some processes were significantly regulated in only one sample. For example, dysregulation of cell death and programmed cell death were detected only in S3.

Most of the selected pathways and GO terms predicted by our algorithm were shared by all four Alzheimer’s disease samples (Figure 2B and D), indicating that this algorithm can be used to predict biological processes and pathways in cells or tissues based on their miRNA expression profiles.

### Evaluation of nanomaterial cytotoxicity using miRNA expression profiles based on deep sequencing data

Due to their unique properties and diverse application in the life sciences, nanomaterials have attracted considerable interest recently [43-45]. However, knowledge of the cellular effects of nanomaterials, such as cytotoxicity, is limited compared with the rapid increase in biological and medical applications [6]. The lack of reliable methods to assess the overall cellular effects of nanomaterials as opposed to consideration of conventional toxicity assays remains a crucial challenge. MiRNAs have been shown to repress gene expression at the post-transcriptional level and to participate in a wide range of cellular processes. Moreover, it is conceivable that miRNAs participate in the cytotoxic activity of nanomaterials, such as apoptosis-like cell death [46]. Combined with the higher stability of miRNA relative to that of mRNA, investigation of miRNA expression profiles represents a beneficial technique for elucidation of the biological effects and the biocompatibility of nanomaterials.

**Figure 4:**
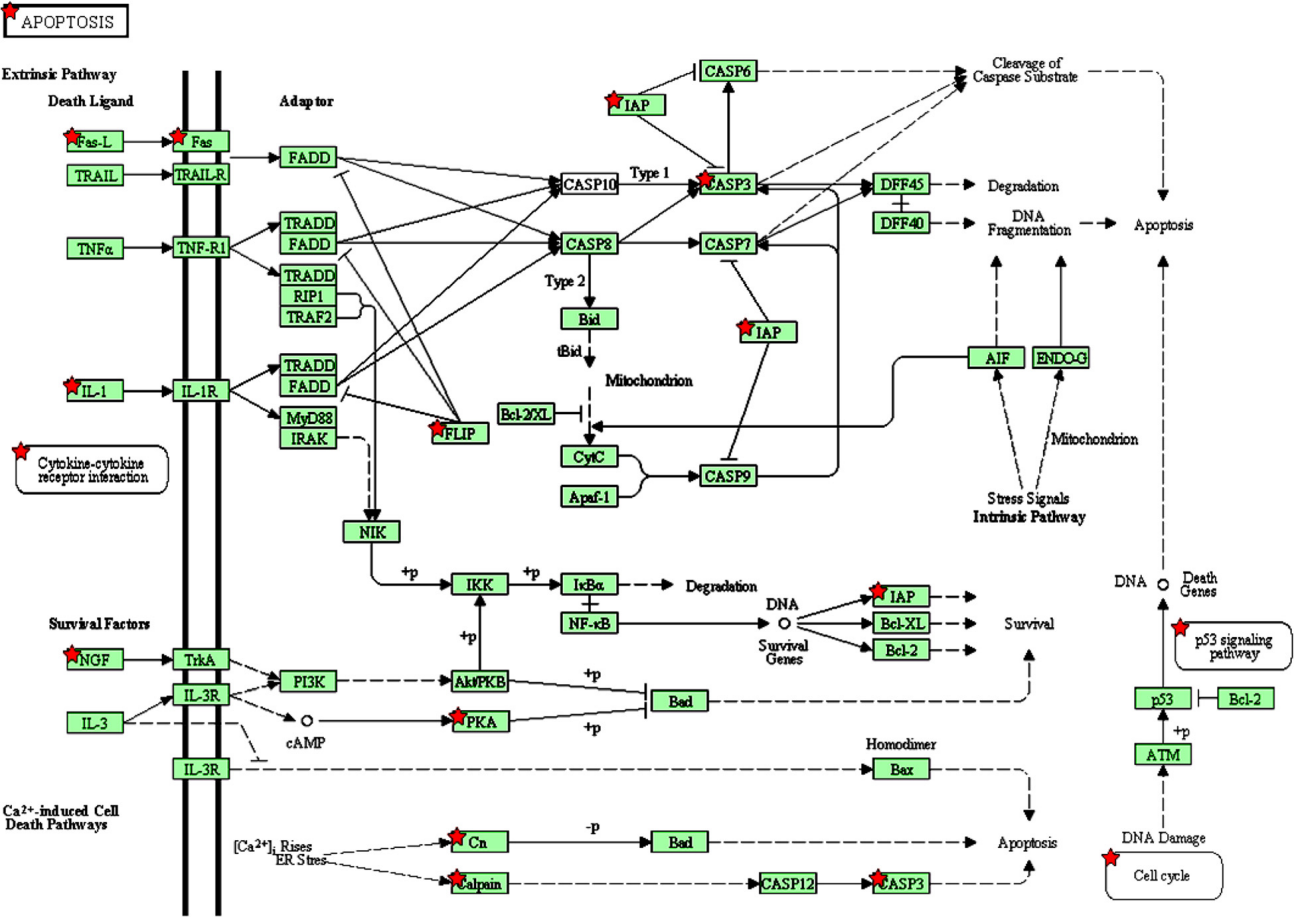
Biological processes in GO terms significantly regulated by miRNAs after CdTe QD treatment.

Previously we have reported that miRNAs may participate in the cytotoxicity of cadmium telluride quantum dots (CdTe QD) [6,16]. The expression patterns of miRNAs were extensively affected after CdTe QD treatment, resulting in apoptosis-like cell death. SOLiD sequencing based miRNA expression profile data were applied to our algorithm. Selected mRNAs indicated to be under significant repression by miRNA profiling were enriched using KEGG pathways and GO terms. KEGG pathways significantly regulated after CdTe QD exposure are shown in Figure 3A. The top pathway on this list was “apoptosis” *(P <* 0.001). Figure 4A summarizes the factors involved in apoptosis pathways. According to this diagram, CdTe QD treatment induced cell apoptosis via the caspase-3 pathway. Other factors including Fas, IL1 or calcium-related pathways may participate in regulating capsase-3. Furthermore, this diagram also displays a cell self-protection mechanism against apoptosis mediated via the inhibitors of apoptosis (IAP) pathway (Figure 4A).

The functions of the mRNAs selected as significantly regulated genes by miRNA profiling with GO analysis were then annotated. As shown in Figure 3B, at the biological process level 4, the top five processes induced by CdTe QDs were: negative regulation of growth, regulation of developmental growth, negative regulation of developmental growth, regulation of cell motion and negative regulation of developmental growth. Other processes included the regulation of cell death, regulation of programmed cell death, negative regulation of cell migration, apoptosis and endocytosis (Figure 3B).

In KEGG pathways and GO terms analyses, one of the main effects induced by CdTe QDs was apoptosis-like cell death and the apoptosis-related proteins were shown to be modulated. According to KEGG pathway analysis, apoptosis proteins were significantly regulated and GO terms analysis annotated CdTe QDs exposure may arrest cell growth and induce apoptosis, which is in accordance with other reports that CdTe QDs induce cell death in variety of cell types [45,47-49].

MiRNAs play a major role in cellular biological processes, including viability, proliferation, development and differentiation. Due to the stability of miRNAs during analysis and the development of sequencing methods, miRNA expression profiling identified as a reliable method for investigation of mRNAs, proteins and pathways involved in particular biological processes. In this study, we proposed a new method for validation of miRNA microarray or deep sequencing results for prediction of proteins and pathways under regulation. Our findings may contribute to early diagnosis of disease and assessment of the biological safety of new materials and drugs.

## Conclusion

MiRNAs play a major role in cellular biological processes, including viability, proliferation, development and differentiation. Due to the stability of miRNAs during analysis and the development of sequencing methods, miRNA expression profiling identified as a reliable method for investigation of mRNAs, proteins and pathways involved in particular biological processes. In this study, we proposed a new method for validation of miRNA microarray or deep sequencing results for prediction of proteins and pathways under regulation. Using our method, the pathways and biological processes involved in Alzheimer’s disease were predicted with high correlation in four independent samples. Moreover, this method was successfully used for annotation of miRNA expression profiles from deep sequencing data for evaluation of CdTe nanomaterial cytotoxicity. As a result, apoptosis pathways were selected as the top pathways involved in CdTe nanoparticle treatment. Our findings may contribute to early diagnosis of disease and assessment of the biological safety of new materials and drugs.

## Abbreviations

GO: Gene ontology; 3′: UTR 3′untranslated region; CdTe: QD cadmium telluride quantum dots; IAP: Inhibitors of apoptosis; GEO: Gene expression omnibus.

## Competing interests

We declare that there are no competing interests.

## Authors’ contributions

BS, NPH, ZDX conceived the project. BS, ZDX designed and implement the algorithm and performed the analysis. FY, FHH contributed to the computational analysis. BS, ZDX wrote the paper. All authors read and approved the final manuscript.
